# In this month’s *Bulletin*

**DOI:** 10.2471/BLT.15.001115

**Published:** 2015-11-01

In the editorial section, Veronica Magar (743) argues that the sustainable development goals need to respond to a range of inequalities, including gender. Taye Balcha et al. (742) draw on the Ethiopian experience to stress the importance of country ownership and local innovations to achieve these goals. 

In the news section, Atasa Moceituba and Monique Tsang report on the health effects of global warming in Fiji (746–747). Christiana Figueres talks to Andréia Azevedo Soares about climate change mitigation ahead of the 21^st^ session of the Conference of the Parties to the UN Framework Convention on Climate Change (748–749). 

## Bangladesh

### Information technology for the health sector

Sheik Mohammed Shariful Islam & Reshman Tabassum (806–809) describe the implementation of information and communication technologies for health. 

## Burkina Faso

### Getting bed nets where they are needed

Valérie R Louis et al. (750–758) study the effects of a first national insecticide-treated bed-net campaign. 

## China

### Planning the tuberculosis response 

Hsien-Ho Lin et al. (790–798) model the potential impact of control measures. 

### Measures to combat drug resistance

Christopher Fitzpatrick et al. (775–784) examine the cost-effectiveness of a programme for drug-resistant tuberculosis. 

## Ethiopia, Kenya, Madagascar, Mozambique, Rwanda and the United Republic of Tanzania

### Preventing complications of childbirth

Linda Bartlett et al. (759–767) observe compliance with guidelines for the active management of the third stage of labour. 

## India

### Right to water in slums 

Ramnath Subbaraman & Sharmila L Murthy (815–816) describe obstacles to water access in Mumbai. 

## Malawi

### Contraception and patient-held records

Aisha NZ Dasgupta et al. (768–774) test family planning cards as a method to evaluate contraceptive use. 

## Philippines

### Health risks in mass gatherings 

Allison E Gocotano et al. (810–814) describe how security measures increased people's exposure to cold weather during the visit of Pope Francis. 

## Global 

### Getting the evidence on medical travel 

Kai Ruggeri et al. (785–789) argue that better evidence is needed for medical travel policies. 

### Access to hepatitis C medicines

Danny J Edwards et al. (799–805) analyse the options for equitable access to new medicines. 

